# Effect of Oral Pregabalin Premedication on Post-Operative Pain in Laparoscopic Gastric Bypass Surgery

**DOI:** 10.5812/aapm.4300

**Published:** 2012-07-10

**Authors:** Mahzad Alimian, Farnad Imani, Seyyed Hamid-Reza Faiz, Alireza Pournajafian, Seyedeh Fatemeh Navadegi, Saeid Safari

**Affiliations:** 1 Department of Anesthesiology, Iran University of Medical Sciences (IUMS), Tehran, Iran

**Keywords:** Laparoscopy, Gastric Bypass, Pregabalin, Pain, Postoperative

## Abstract

**Background:**

Post-operative pain and the administration of opioids to relieve it, is considered to be one of the important issues in surgery wards. This issue is even more significant in obese patients, because of the side effects of opioids. Pregabalin is an analog of gamma aminobutyric acid (GABA) which can be effective in dealing with post-operative pain.

**Objectives:**

This study will consider the effect of oral pregabalin in relieving the pain of obese patients after gastric bypass surgery.

**Patients and Methods:**

In a double blind clinical trial, 60 candidates for laparoscopic gastric bypass surgery were enrolled in the study through convenience and non-random sequential sampling, into two groups; pregabalin group and control group. Inclusion criteria consisted of: morbid obesity with a body mass index (BMI) > 35, age 18–50, American Society of Anesthesiologists (ASA) status I or II, and willingness to take part in the study. Patients in the pregabalin group received 300 mg of oral pregabalin on the morning of the surgery. Post-operative pain was controlled by the patient-controlled intravenous analgesia (PCIA) method, an AutoMed infusion pump containing 20 mg of morphine and normal saline (total volume 100 cc) was administered to all patients after surgery. Patients’ level of pain were compared by considering their pain intensity on a visual analog scale (VAS), and the occurrence of nausea/vomiting from recovery, until 24 hours after surgery.

**Results:**

A total of 60 patients were compared; 30 patients in each of the pregabalin and control groups. Both groups were similar in age and sex distribution. Mean pain intensity levels during the whole follow up were lower in the pregabalin group than in the control group, up to a maximum of 24 hours after the operation (*P* < 0.001). Incidence of nausea/vomiting was greater in the control group than in the pregabalin group (*P* < 0.001).

**Conclusions:**

The findings of this study indicate that oral pregabalin (300 mg dose) can alleviate patients’ pain and nausea/vomiting and notably reduce adverse effects.

## 1. Background

Nowadays, the number of people undergoing surgery in order to lose weight and control the complications of morbid obesity is increasing. As a result of the administration of opioids to relieve post-operative pain, respiratory and cardiovascular side effects in laparoscopic gastric bypass patients, are the most important issues to be addressed. Therefore, establishing analgesia and at the same time preserving respiratory and cardiovascular stability, have significant roles to play in the treatment of these patients. Administering non-opioid drugs to alleviate pain in these patients and reducing the dose of intravenous opioids, are among the most effective methods in controlling post-operative pain and its side effects (1–3).

Pain during and after surgery can lead to sensitization and consequently over-sensitivity to pain, it can also transform post-operative acute pain into chronic pain (4). Relieving pain during an operation by administering opioids is a common practice, which can also result in undesirable side effects. In order to scale down these side effects, other non-opioid drugs can be utilized.

At present, patient-controlled intravenous analgesia (PCIA) is extensively employed in surgery wards as a way to deal with post-operative pain. In this method infusion pumps are used, these tools allow patients to receive a prescribed dose of analgesic substance intravenously. Opioids are commonly used in this method. A number of limitations accompany the benefits of this practice; the cost is high and administering opioids increases the side effects and risks of abdominal surgeries (5–7).

A solution for acute post-operative pain is utilizing compounds pre-emptively, which alleviates pain by reducing sensitivity and lowering the chances of acute post-operative pain turning into chronic pain.

Among these compounds are anti-epileptic drugs. Anti-epileptic drugs like carbamazepine have been utilized as an adjuvant treatment for chronic pain complications for years. Therefore, it was no surprise that pregabalin (derivatives similar to gabapentin) can be used to relieve pain in a range of different diseases, including pain syndromes (8–10).

Pregabalin is an anticonvulsant drug that reduces calcium entry to the nerve terminals of the central nervous system and lowers substance P, glutamate and noradrenalin levels, which play roles in creating a sense of pain (11).

Pregabalin is also utilized in the reduction of neuropathic and even inflammatory pain, tissue irritation and fibromyalgia (12–16). Initial findings suggest that pregabalin can be effective in controlling acute post-operative pain, including major operations (abdominal, hysterectomy and orthopedic surgery). Pregabalin can considerably reduce pain intensity after an operation and lower the required dose of opioids. However, some studies point out that pregabalin lacks the necessary effectiveness in major operations and its effect is limited to minor operations (17).

Studies also suggest that gabapentin has a satisfactory effect in alleviating post-operative pain (18, 19). Pregabalin has been introduced as the new gabapentinoid with a higher efficacy and more desirable pharmacological profile than gabapentin. Therefore, it seems that pregabalin could be a better choice in alleviating post-operative pain than gabapentin.

## 2. Objectives

The aim of this study was to evaluate the effects of 300 mg oral pregabalin, before laparoscopic gastric bypass surgery, on pain intensity measured by a visual analog scale (VAS), and the incidence of post operative nausea/vomiting.

## 3. Patients and Methods

This study was designed and carried out as a double blind, non-randomized clinical trial. After approval by the center’s medical ethics committee and obtaining informed consent from all of the candidates for laparoscopic gastric bypass surgery, patients in the pregabalin group received 300 mg of pregabalin before entering the operation room. Sampling was in convenience and non-random sequential form. The control group was an historical control, which was adapted from another study that was conducted almost at the same time, with the same criteria as this study and the method of data collection was also the same. Inclusion criteria consisted of; morbid obesity with a body mass index (BMI) > 35, age 18–50, an American Society of Anesthesiologists (ASA) status of I or II and a willingness to take part in the study. Exclusion criteria consisted of; history of hypersensitivity to pregabalin or derivative, hereditary problems with galactose and glucose, lactation, blood pressure less than 90 mm Hg, history of addiction, or moderate to severe respiratory disorder.

In the pregabalin group 300 mg of pregabalin was given to the patients an hour before their operation. The PCIA method with morphine content was utilized to control the pain following surgery in both groups.

After establishing an intravenous line and monitoring, 3 cc/kg of Ringer’s lactate solution was administered, and 2 mg of midazolam and 2 μg/kg of fentanyl were used as a premedication. The patients were given 5 mg/kg of sodium thiopental and 0.15 mg/kg of cisatracurium for induction and then they were intubated. For maintenance of anesthesia, isoflurane (1%) and remifentanil (0.2 μ/kg/min) were administered. During the operation cisatracurium (2 mg) was given every 30 minutes. Twenty minutes before the end of the operation, remifentanil administration was stopped and 2 cc of fentanyl was injected.

If the patient’s blood pressure exceeded 180/100 mm Hg during the operation, trinitroglycerin (TNG) was administered in 5 μg/min incremental doses to lower the blood pressure below 160/90 mm Hg.

The anesthesiologist had no information concerning the drugs given to the patients before the operation. Patients were checked against a VAS upon recovery and 4, 12 and 24 hours after the operation. They were also checked in regard to side effects of nausea and vomiting until the end of the first 24-hour period. Pain control in the pregabalin and control groups was according to the acute pain protocol and through PCIA (AutoMed pump, ACEMEDICAL AM3400, South Korea) containing 20 mg of morphine in normal saline with a total volume of 100 cc which was administered continuously in 5 cc per hour and 2 cc bolus injection with a 30 minute lockout interval.

The data were collected by questionnaires and analyzed through statistical analysis software (SPSS 17). Quantitative data was presented as mean and standard deviation, and qualitative data was presented as frequency. In order to compare quantitative and qualitative data between the two groups (in case of adherence to normal distribution) K-2 assessment was utilized for the qualitative variables and *t*-test for quantitative variables. In cases of non-adherence, non-parametric equivalent assessment was employed. Significance level was considered as *P* < 0.05 in this study.

## 4. Results

A total of 60 patients were divided into two groups of 30; pregabalin group and control group. The mean age of the patients was 33.8 ± 7.6 in the pregabalin group and 37 ± 5.2 in the control group. Although the mean patient age was slightly higher in the pregabalin group, the difference was not statistically significant (*P* = 0.70). Considering sex distribution, it is noted that women comprised the majority of the patients. In the pregabalin group 25 patients (84%) and in the control group 23 patients (76.7%) were female. The difference in sex distribution between the two groups was not statistically significant (*P* = 0.432).

Pain intensity on VAS in the pregabalin and control group at the time of recovery is presented in *Table 1*. The difference in mean pain intensity on VAS is 1.46 ± 0.3 between the two groups, which is statistically significant (*P* < 0.001). That is, the pain intensity in the patients of the pregabalin group was significantly lower than in the control group at the time of recovery. Based on *figure*, the mean pain intensity 4, 12 and 24 hours after the operation was lower on average in the pregabalin group (*P* < 0.001). In order to compare the mean variance in the pain intensity between the two groups, frequent measurements taken at different times were carried out and the variance analysis of the frequent measurements was considered to eliminate differing factors such as time, age and sex. According to the analysis significant changes which occurred during the effective period, stemmed from the treatment pack type (administered drugs) (*P* < 0.001) and the time factor. Patients’ age (*P* = 0.545) and sex (*P* = 0.136) had no effect on the changes.

**Figure.**
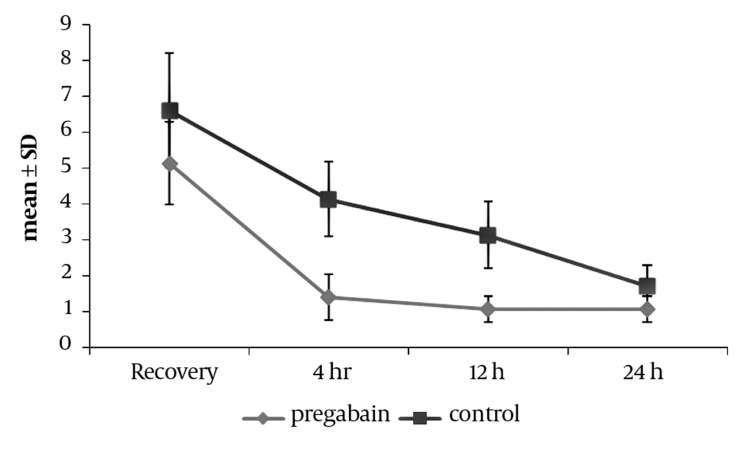
Distribution of VAS in Patients of Pregabalin and Control Groups During the Follow-Up Period After the Operation

**Table 1:** Comparison of Pain Intensity between Two Groups

Pain Intensity VAS^a^	Recovery	4 Hours After the Operation	12 Hours After the Operation	24 Hours After the Operation
Pregabalin group, Mean ± SD	5.1 ± 1.1	1.3 ± 0.6	1.1 ± 0.4	1 ± 0.4
Control group, Mean ± SD	6.6 ± 1.5	4.1 ± 1	3.1 ± 0.9	1.7 ± 0.6
*P* value	< 0.001	< 0.001	< 0.001	< 0.001

^a^Abbreviation: VAS, visual analog scale

Incidence of nausea/vomiting in the two groups is given in *Table 2*. All patients in the control group complained about nausea/vomiting. Nausea occurred in 17 people (56.7%) and vomiting occurred in 13 people (43.3%). In the pregabalin group only 6 people (20%) complained about nausea/vomiting, 4 patients (13.3%) suffered nausea, and 2 patients (6.7%) vomiting. Statistically, nausea/vomiting were significantly more frequent in the control group than in the pregabalin group (*P* < 0.001).

**Table 2:** Comparison of Nausea and Vomiting Incidence Between Two Groups

	Nausea, No. (%)	Vomiting, No. (%)
Pregabalin group	4 (13.3)	2 (6.7)
Control group	17 (56.7)	13 (43.3)
*P* value	< 0.001	< 0.001

## 5. Discussion

The findings of this study indicate that a single 300 mg dose of pregabalin before the operation can significantly alleviate the pain intensity of laparoscopic gastric bypass patients, who are treated with morphine analgesia by PCIA. It also reduces the incidence of nausea/vomiting after the operation.

Parallel to the increasing growth of obesity, surgical interventions have progressed to control weight, and reduce the side effects of obesity such as; diabetes, blood pressure, cardio and respiratory diseases, arthritis, etc. (1, 3). This progress also includes gastric bypass surgery, which nowadays, is usually laparoscopic, and it is favored more than other methods.

The usual practice for alleviating post-operative pain is the administration of long acting opioids, especially morphine. As well as advantages such as; an extended analgesic period and lower costs for the patient, this practice can also lead to side effects such as; a reduced level of consciousness, depression of the respiratory system, nausea/vomiting, constipation and even a prolonged period of hospitalization (5–7).

Patient-controlled analgesia has been employed in clinical wards to effectively relieve pain following operations; however, since opioids are still administered by this method, to lower the dose of opioids delivered, other methods need to be considered as well. In recent years the administration of pregabalin to relieve post-operative pain has been favored. Like gabalin, pregabalin has anticonvulsant, analgesic and anti-anxiety characteristics, but it differs from gabalin in possessing better pharmacokinetics such as dose-independent absorption which results in its preference over gabalin in clinical practice (13, 14).

Administering pregabalin to relieve the post-operative pain of laparoscopic operations has been reported in a number of studies. Agrawal *et al.* gave patients a 150 mg dose of pregabalin per hour, before a laparoscopic cholecystectomy under general anesthesia to relieve pain. Although in this clinical trial intravenous fentanyl was injected in both groups, the pain intensity was significantly lower in the group receiving pregabalin, than in the control group (8). The dose of pregabalin in the present study was 300 mg, which indicates that this amount can be tolerated by patients without any particular side effects, and it alleviated their pain better when compared to the control group.

In addition, in two other studies by Jokela R *et al.* on laparoscopic gynecological surgery, various pregabalin doses were compared (150, 300 and 600 mg) (20, 21). These two studies indicate that administering 300 mg of pregabalin before an operation and repeating it 12 hours after the operation brings about remarkable effects in relieving pain, but at this dosage level some side effects including dizziness appear more frequently. Administering a dose of less than 300 mg, did not result in a satisfactory analgesic effect. For example, Chang *et al.* administered two separate doses of 150 mg, before and 12 hours after an operation, but they did not find a significant difference in post-operative pain intensity between the two groups (19). Looking into various research studies, there are also studies that question the efficacy of pregabalin, especially in painful surgeries. For example Mathiesen *et al.* also examined the effect of administering 300 mg of pregabalin an hour before an operation on the post-operative pain. They carried out two different research studies on patients who had undergone painful surgeries including, hip arthroplasty and abdominal hysterectomy. No statistically significant difference was found between the two groups, pregabalin and placebo (22, 23).

A meta-analysis in 2011 indicated that administering pregabalin does not result in less pain during the first 24 hours following surgery (24). However, doses of opioids after receiving pregabalin were significantly decreased during the 24-hour period following the operation. In this present study, a 300 mg single dose of pregabalin before the operation significantly relieved the pain of patients who had undergone laparoscopic gastric bypass surgery, compared to the control group. It also lowered the frequency of nausea/vomiting in these patients after the operation without any side effects. According to the type of surgery, it seems that this dose is suitable for major surgeries to be used with opioids.

Non-random selection, and an insufficient number of patients volunteering at the treatment center that was surveyed, are among the limitations of this present study. Nevertheless, the remarkable efficacy of pregabalin made our study strong enough to demonstrate differences between the two groups. Therefore, it appears necessary for further research with simultaneous random groups and a larger number of patients to be conducted and the exact efficacy level of pregabalin needs to be investigated.
